# Health Disparities in Delirium

**DOI:** 10.1093/geroni/igab046.568

**Published:** 2021-12-17

**Authors:** Robert Dicks, Jimmy Choi, Christine Waszynski, Kadesha Collins-Fletcher, Beth Taylor, Catherine Martinez, David O'Sullivan

**Affiliations:** 1 Hartford Hospital, West Simsbury, Connecticut, United States; 2 Institute of Living, Institute of Living, Hartford, Connecticut, United States; 3 Hartford Hospital, Hartford, Connecticut, United States; 4 Evernorth, Saint Louis, Missouri, United States; 5 Vanderbilt University, Nashville, Tennessee, United States; 6 Hartford Healthcare, Hartford, Connecticut, United States

## Abstract

Racial and ethnic minority populations in the US experience greater cumulative disease burden, as well as social and economic barriers, stressors, and limited advocacy/access to culturally informed healthcare. This increased risk burden is expected to be associated with an increased risk for delirium during acute care encounters. Previous studies on health disparity and delirium are limited and report equivocal findings regarding delirium incidence, possibly related to sample bias or non-validated measures. Risk for delirium during acute care in health disparity populations (HDP) that include Black African Americans (BAA) and Hispanic-Latinx (HL) has not been systematically studied using validated measures. We conducted a retrospective analysis utilizing our delirium program (ADAPT) registry that systematically assessed all hospitalized patients through their entire hospital stay for the years 2018-2019 (36K patients, 80% NHW, 11% HL, 9% BAA). The Confusion Assessment Method (CAM and CAM-ICU) and Richmond Agitation Sedation Scale (RASS) were used as screening assessments to identify delirium. We know from previous studies that negative CAM results in our environment have high specificity. The incidence of delirium between populations was compared using a chi-square test. Delirium incidence was higher in HDP (BAA combined with HL) compared to NHW in 71-80yo (16.0% vs 12.6%, p=0.003). Delirium incidence was not different in all other age groups compared; <65yo (p=0.191), 61-70yo (p=0.223), 81-90yo (p=0.644). Understanding the association, or lack thereof, between health disparities, ethnic and race-based risks for delirium is expected to provide important insights into more focused delirium assessment, prevention and mitigation strategies in these populations.

